# Main-Duct Intraductal Papillary Mucinous Neoplasm Complicated by a Pancreaticogastric Fistula and a Pancreaticocholedocal Fistula

**DOI:** 10.7759/cureus.38502

**Published:** 2023-05-03

**Authors:** Sarvanand Patel, Umaima Al Salmi, Khalid Al Shamousi

**Affiliations:** 1 Department of Internal Medicine, Cedars Sinai Medical Center, Los Angeles, USA; 2 Department of Medicine, Sultan Qaboos University Hospital, Muscat, OMN; 3 Department of Medicine, Sultan Qaboos University, Muscat, OMN

**Keywords:** intraductal papillary mucinous neoplasm (ipmn), pancreatic cancer prevention, pancreatic cancer resection, pancreaticogastric fistula, intraductal papillary malignancy, intraductal papillary neoplasm of bile duct, endoscopy ercp, biliary stent cytology, external pancreatic fistula, biliary tract fistula

## Abstract

Intraductal papillary mucinous neoplasms (IPMNs) of the pancreas are a spectrum of benign to malignant epithelial neoplasms that are characterized by papillary proliferation, duct dilation, and cyst formation. A rare complication of IPMNs is fistula formation into adjacent organs such as the duodenum, biliary system, and stomach. Here, we report a case of IPMN with a pancreaticobiliary fistula and pancreaticogastric fistula. An 84-year-old woman with early Alzheimer’s disease was diagnosed with IPMN of the pancreas. She deferred surgery given her age and remained asymptomatic for five years until presenting with cholangitis. She had been further evaluated and was found to have a pancreaticobiliary fistula, which was treated with biliary stent placement. Her subsequent admission involved the formation of a new pancreaticogastric fistula. This case highlights potential complications of excess mucin production from unresected IPMNs and demonstrates a guarded prognosis in elderly patients.

## Introduction

Intraductal papillary mucinous neoplasm (IPMN) was first reported in 1982 as a “mucin-producing tumor.” It originates from the epithelium of the pancreatic duct, characterized by papillary growth with thick mucin secretion leading to dilatation of the involved pancreatic duct. Anatomically, IPMNs are classified into three types based on the duct involved: the branch duct type (small tumors, commonly adenomas, relatively low incidence of carcinoma); the main duct type (more proliferative, larger, complex tumors with atypia); and the mixed type, which includes features of both the above types [1].

IPMN can be asymptomatic or present with episodic pancreatitis, abdominal pain, jaundice, or weight loss. The incidence is not known; yet, the number of cases has increased due to improvements in imaging modalities [2]. Concerning features of probable malignancy can be seen on imaging, which include obstructive jaundice with a cystic lesion, enhanced solid components, and a main pancreatic duct (MPD) size ≥10 mm. Worrisome features of carcinoma include duct size 5-9 mm and cyst size ≥30 mm [3].

A leading feature of many IPMNs is excessive mucin production. It has been demonstrated that most IPMNs produce mucin 2 (MUC2) while mucin 1 (MUC1) is not expressed, except in those cases that showed an invasive tubular component resembling ductal carcinoma [4]. In the early phase of IPMN, epigastric discomfort or pain can be related to meals due to the hyperproduction of mucin that obstructs normal pancreatic secretions. While, in the later phase, it can be associated with the development of obstructive jaundice and cholangitis with pancreatobiliary fistula formation [3]. This is an infrequent occurrence and fistulization can occur in organs such as the stomach, duodenum, bile duct, colon, and small intestine. This can even be seen in benign IPMN [5].

Here, we present a rare case of a main-duct IPMN that presented initially with a pancreaticogastric fistula and later the formation of a pancreaticobiliary fistula.

## Case presentation

Our patient is an 89-year-old woman with a history of early Alzheimer's, hyperlipidemia, and osteoporosis. Her initial symptoms included weight loss, abdominal fullness, and changes in stool characterization to a liquid of putty color. She had no significant history of smoking or alcohol use. A CT scan four months later from symptom onset showed an ectatic cystic prominence and a focal cystic lesion involving the proximal pancreas and the region of the pancreatic head measuring 28 mm x 23 mm with suspected marked dilatation of the proximal pancreatic duct to greater than 20 mm. MRCP showed four distinct cystic lesions (2 saccular dilatations in the distal main pancreatic duct measuring 23 mm and 2 adjacent cysts in the pancreatic head measuring 28 mm), and the pancreatic body was dilated to 0.4 cm concerning for IPMN. Esophagogastroduodenoscopy (EGD) at this time showed mucus extravasation from the major papilla, with cyst fluid showing carcinoembryonic antigen (CEA) of 9500 and cancer antigen 19-9 (Ca19-9) of 2741. The patient declined surgery given her age and chose to follow with serial imaging and symptomatic management. An endoscopic ultrasound (EUS) six months later showed a 56 mm cystic lesion in the neck of the pancreas (the earlier two cysts) with early degeneration, septations, and a mural nodule. At this time, the patient continued to defer surgical management.

At the one-year follow-up, her MRI showed dilation of the main duct at 19 mm from 4 mm and dilated branch ducts. She had signs of biliary dilatation at this time with no symptoms of obstruction. After three years, she continued to be largely asymptomatic with MRI showing a stable pancreatic cyst size, however, it was noted her tail pancreatic duct increased to 26 mm x 32 mm from 16 mm x 27 mm.

**Figure 1:**
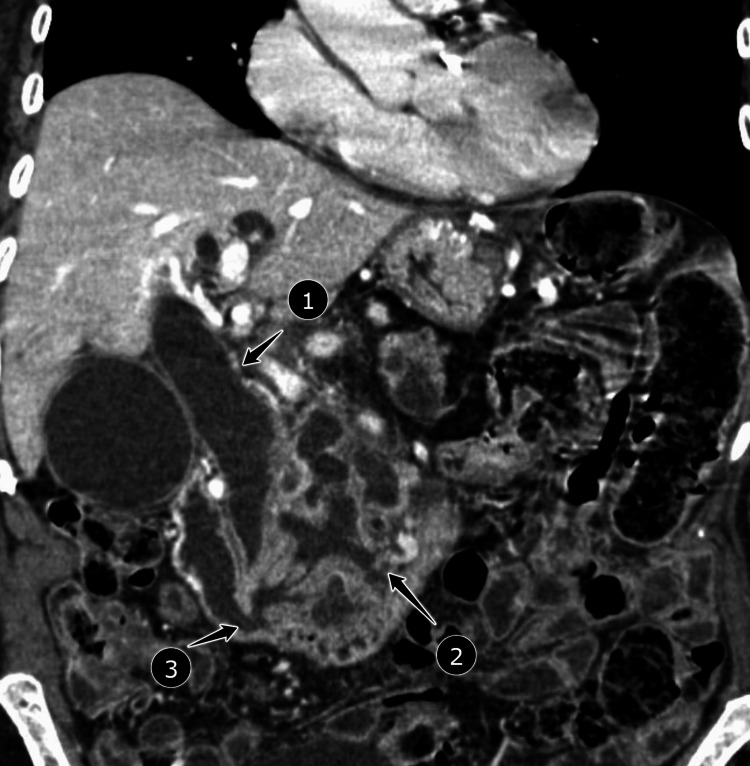
Markedly dilated gallbladder with a very large multicystic mass involving the pancreatic head and extending into the stomach with communication of the pancreatic duct into the stomach. There is significant obstruction of the common bile duct. 1. Dilated common bile duct due to mucin congestion. 2. Dilated main and side branches of the pancreatic duct due to mucin congestion. 3. Area of the ampulla of Vater

**Figure 2:**
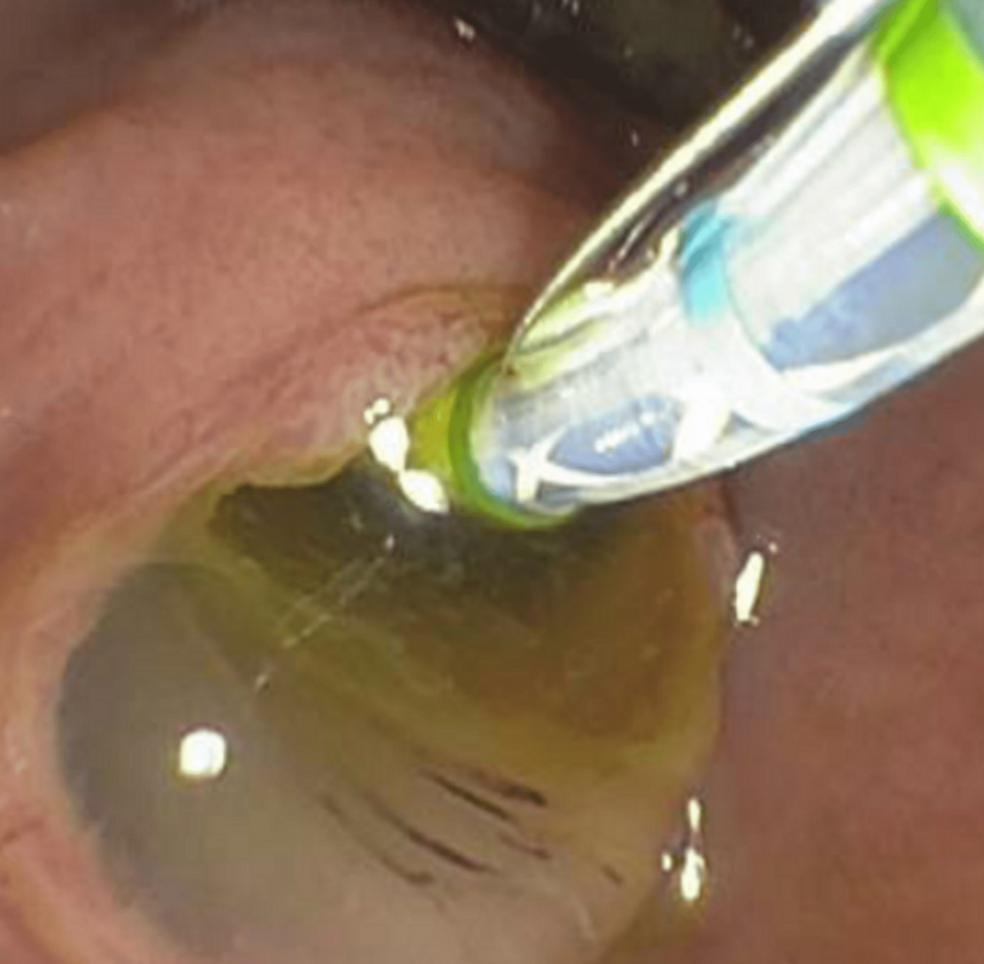
Initial EGD with extruded mucus was seen in the stomach through a fistula opening. Area of the papilla during ERCP with a fish mouth appearance during biliary cannulation EGD: esophagogastroduodenoscopy; ERCP: endoscopic retrograde cholangiopancreatography

**Figure 3:**
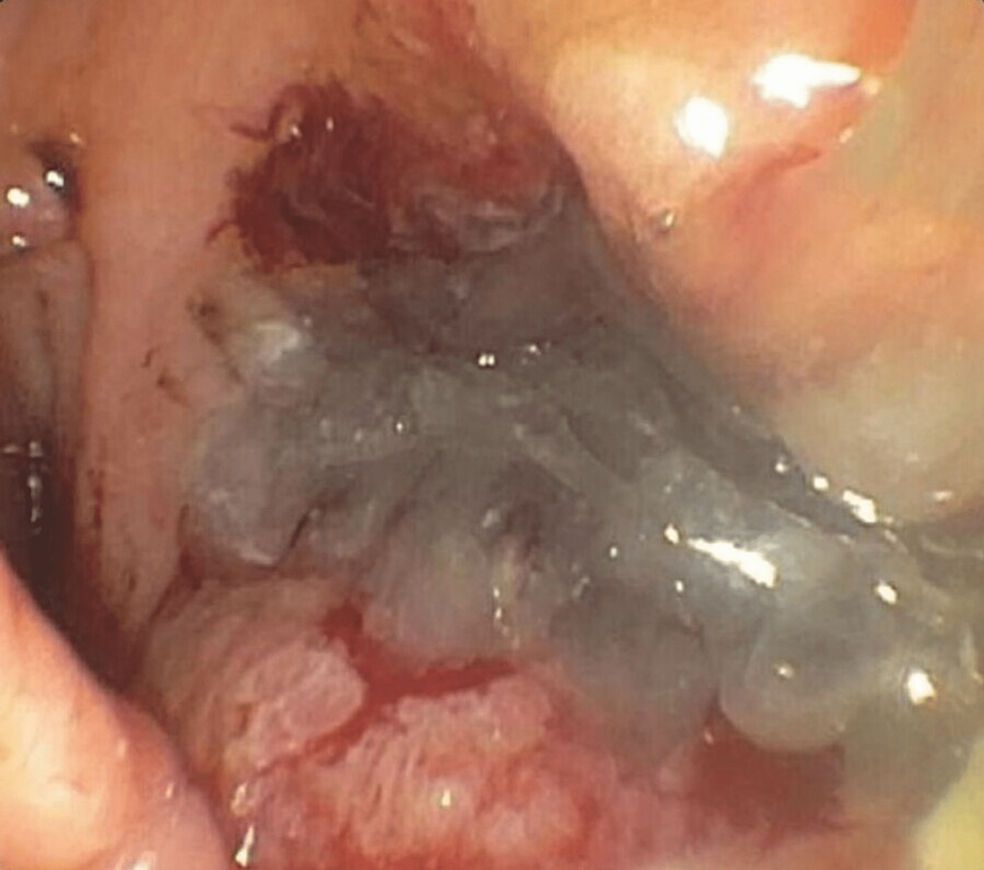
Initial EGD with extruded mucus was seen in the stomach through a fistula opening EGD: esophagogastroduodenoscopy

Eventually, five years post-diagnosis, the patient presented with elevated bilirubin and isolated fevers. Her common bile duct (CBD) showed bile duct elevation to 1.9 cm on abdominal US. Endoscopic retrograde cholangiopancreatography (ERCP) showed a massively dilated CBD, with a pancreaticogastric fistula draining mucus into the stomach (Figures 1, 2). At this time, the patient was no longer able to give consent, therefore the patient's daughter wanted to proceed with further interventions. A sphincterotomy was performed with four plastic stents, and one biliary stent was placed in the CBD. The patient tolerated the procedure well, and new MRI imaging of the abdomen after ERCP showed decreased pancreatic ductal dilatation, with a visualized communication to the stomach with the pancreatic duct both proximally and distally from the head and body, as well as multiple new outpouchings of the stomach and duodenum with the suggestion of fistulous connections between each of several additional smaller cystic areas in this location (Figure 3). The patient was discharged with a follow-up in three months for a stent exchange.

**Figure 4:**
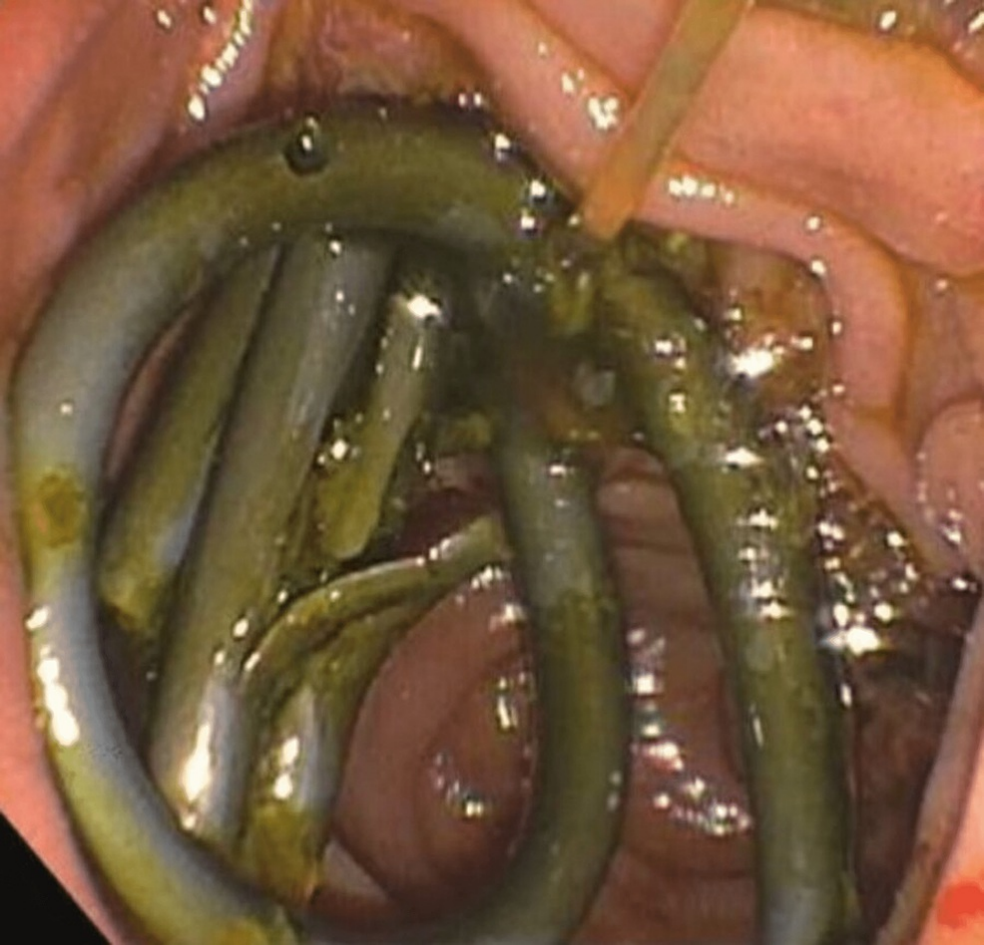
Patient presented with cholangitis after previous stent exchange Five previous plastic stents that were placed were occluded.

One month prior to the planned ERCP, the patient presented to the emergency department with fevers, elevated liver function tests (LFTs), and CT findings of intrahepatic and extrahepatic ductal dilatation, showing cholangitis. ERCP performed showed that the initial stents placed were occluded (Figure 4). The five stents were removed from the biliary tree with a snare, and one biliary stent was placed in the CBD. Biopsies taken at this time from the common bile duct were negative for malignancy.

Two weeks later, she presented again with deranged LFTs. This time, a new spontaneous fistula was identified in the common bile duct. The previous stent was not visualized at its previous insertion (including the pelvic area). The common bile duct also contained filling defects thought to be mucin balls. A coated metal stent with side holes was placed, and the patient was discharged with a resolution of symptoms on antibiotics.

**Figure 5:**
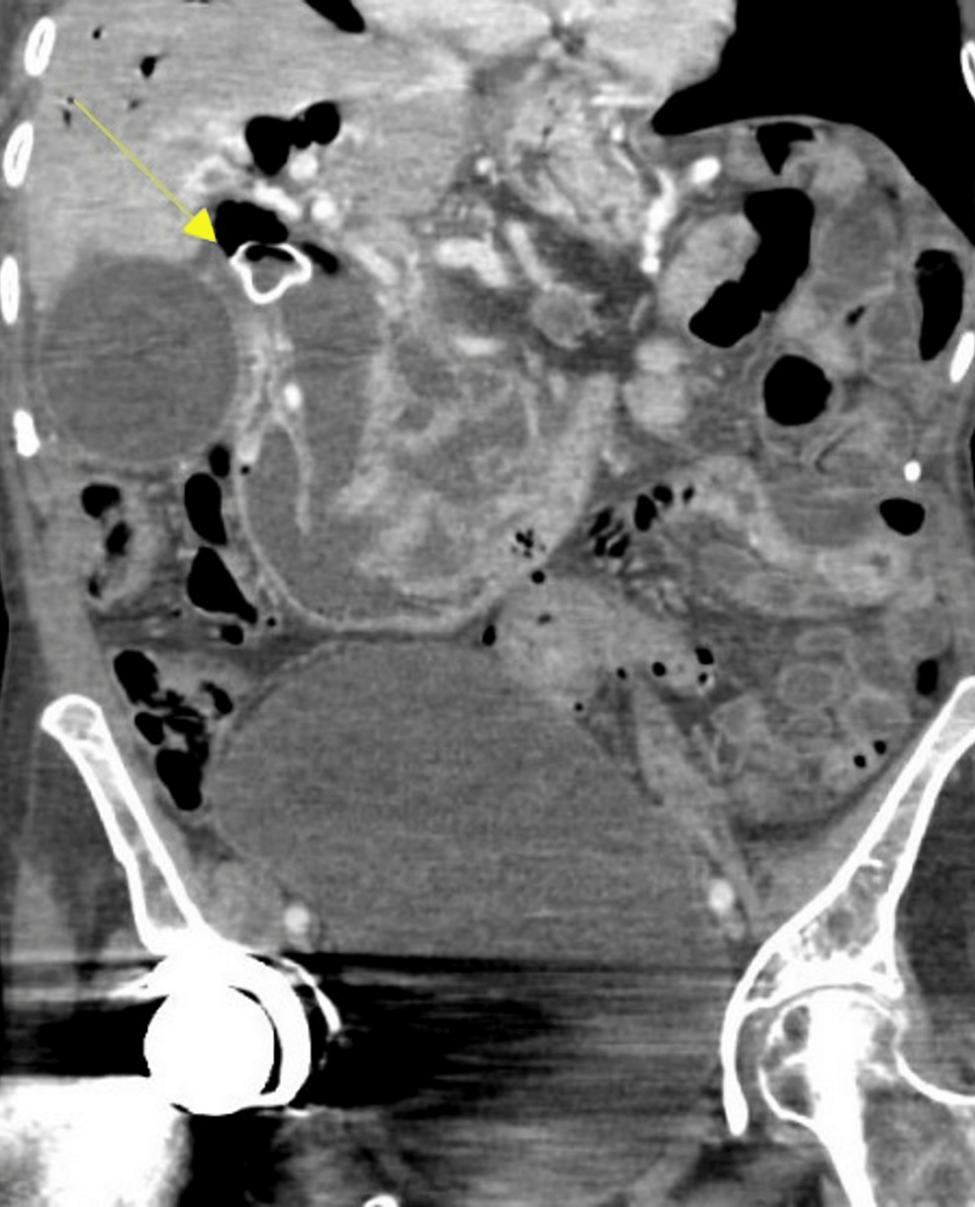
Five months after the initial fistula Interval choledochoduodenostomy with metal stent placement (arrow). Interval development of extensive pneumobilia with increased biliary dilatation.

**Figure 6:**
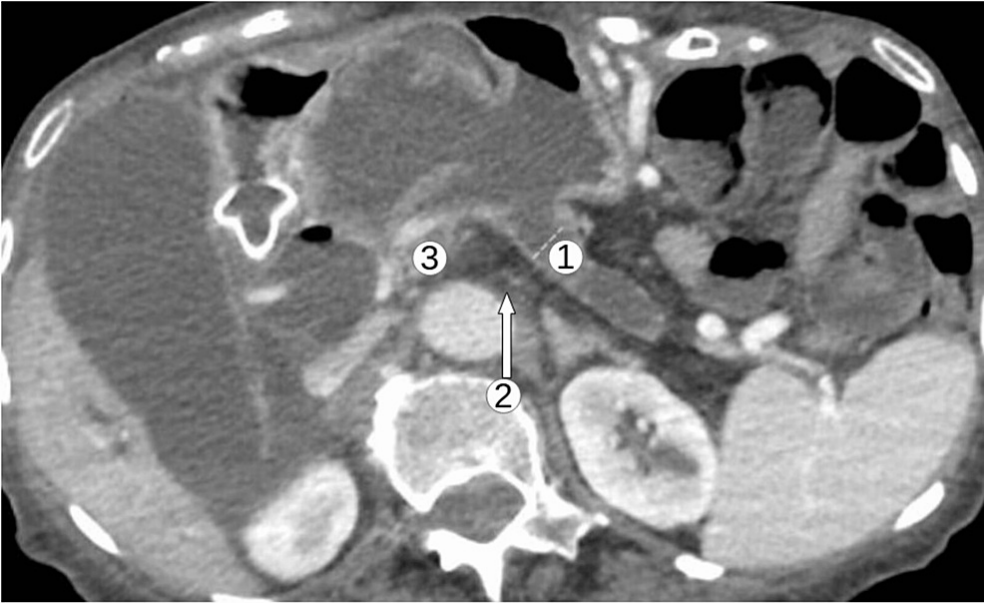
Axial view after choledochoduodenostomy CBD is dilated down to the ampulla. Gas within the gallbladder lumen is noted. Severely atrophic with dilated main pancreatic duct measuring 12 mm with fistula to the stomach and common hepatic duct. 1: Biliary dilatation through the common bile duct. 2: Atrophic pancreas noted. 3: Fistulous connection to stomach visualized. CBD: common bile duct

After her new pancreatic duct to common bile duct (PD-CBD) fistula, our patient's health worsened. She was again admitted one month later for biliary obstruction and cholangitis and underwent a EUS-guided choledochoduodenostomy with a lumen-opposing metal stent (Figures 5-6). During this admission, the patient was also noted to have a failure to thrive given her worsening oral intake, and was briefly placed on total parenteral nutrition (TPN).

**Figure 7:**
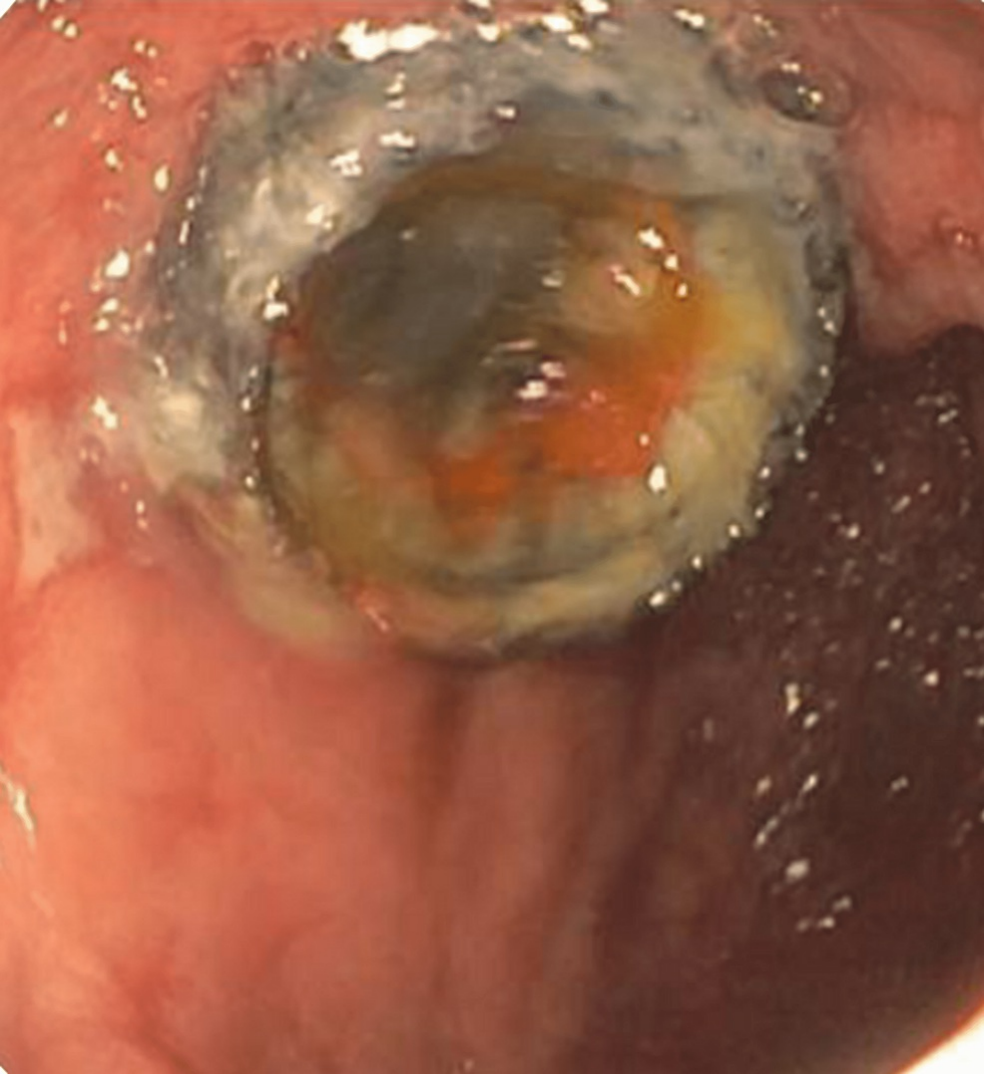
The patient presented with elevated LFTs again EGD and ERCP with mucin suction and removal from both the pancreaticogastric fistula and the bile duct through the choledochoduodenostomy fistula. Here, the cholodochoduedonostomy fistula is visualized. LFT: liver function test; EGD: esophagogastroduodenoscopy; ERCP: endoscopic retrograde cholangiopancreatography

Subsequently, our patient did present to the hospital on four more occasions; two occasions involving a urinary tract infection from her chronic indwelling Foley catheter that was placed due to her dementia and two episodes of cholangitis with stent exchanges (Figure 7). She had also received three additional scheduled stent exchanges as an outpatient. Given her worsening malnutrition causing generalized anasarca, development of Clostridium difficile colitis, and a deep vein thrombosis on her last hospitalization, the patient’s family transitioned her to hospice nine months after the development of her PD-CBD fistula.

## Discussion

Our case demonstrates the rare findings of a pancreaticogastric and a PD-CBD fistula in a patient who refused surgical intervention for IPMN. As IPMN progress slowly, it may involve the formation of a fistula to the adjacent organs [5]. A retrospective analysis of 274 patients with IPMN observed fistula formation in 18 patients (6.6%). Fistula formation into multiple organs was reported in 39% of these and most commonly involved the duodenum (67%), followed by the stomach (44%), bile duct (33%), colon (6%), and small intestine (6%) [6-7]. A case report on fistulization into the thigh has been reported as well [8]. Our case represents a pancreaticobiliary fistula in a patient with IPMN, which is a rare manifestation. It is usually associated with obstructive jaundice and cholangitis, similar to what happened to our patient.

Prior to our patient's fistula formation, a pancreatic duct dilation to 40 mm (MPD diameter of >10 mm) is found to be associated with malignancy, with a specificity of 92% and the presence of a mural nodule, yet she refused surgery given her age [4-5]. Controversy persists regarding optimal duct diameter cut-off for resection and the need for either observation or resection in main duct IPMNs. The European guidelines recommend resection in all fit individuals, the International Association of Pancreatology (IAP) guidelines require the presence of any high-risk stigmata: (A) jaundice due to the IPMN; (B) enhancing mural nodule over 5 mm; or (C) main pancreatic duct dilatation over 10 mm). The American College of Gastroenterology (ACG) recommends referral to a multidisciplinary team discussion, whereas the American Gastroenterological Association (AGA) guidelines do not mention this group with main duct dilatation specifically [9-10]. Pancreaticoduodenectomy and distal pancreatectomy are related to complications in 25% of patients such as anastomotic leakage or stenosis, pancreatic fistula, intra-abdominal abscess, and pancreatitis. In-hospital morbidity is 37%, and in-hospital and 30-day mortality are 1.4% and 2.7%, respectively [11]. Additionally, in the treatment of IPMN with a fistula, the fistula should be removed regardless, to avoid malignant dissemination.

This case demonstrates the potential course of non-surgical surveillance and symptomatic care of IPMN. Her frequency of ED admissions increased and she presented three months later with occluded stents. Also, two weeks later, she presented with a discovered fistula in the common bile duct. The five-year survival of patients after surgical resection for non-invasive IPMN is reported to be at 77-100% [11]. Vanella et al. performed a meta-analysis and found disease-specific mortality of 23 for all IPMN, 32 for MD-IPMN, and five for BD-IPMN per 1000 patient-years [12]. The patient did well with respect to the studied five-year survival without surgery.

Since diagnosis, the patient was largely asymptomatic until five years, when she presented to the ED for complications from excessive mucin production. However, once her initial pancreatic-CBD fistula took place, the patient’s frequency of hospitalizations increased for multiple admissions for cholangitis. The causes of cholangitis in biliary pancreatic fistula are direct invasion by the tumor into the biliary, external compression, or mucin impaction within the lumen [12]. The incidence of obstructive jaundice due to the mucinous material of a pancreaticobiliary fistula associated with IPMN is 97% [12]. Yamaguchi et al. reported that mean survival times in patients with pancreatobiliary fistulas associated with IPMNs who underwent surgical resection versus those who did not undergo surgical resection were 47.9 and 10.4 months, respectively [13]. The causes of death in patients without resections were cholangitis or hepatic insufficiency. Shorter interval surveillance with EUS (3-6 months) is considered in patients, who for reasons of operative risk or personal preference, have chosen heightened surveillance over resection [4]. Stent dislodgement was a complication with our patient and monitoring for stent dislodgment could also have alleviated hospital admissions. This may require earlier stent exchange or stents with side holes to prevent cholangitis.

Our patient’s biliary biopsy was negative for high-grade dysplasia and extension of malignancy in the setting of her obstructive jaundice and cholangitis. Previous studies demonstrate the incidence of obstructive jaundice due to the mucinous material of pancreaticobiliary fistulas associated with IPMNs is 97.1%. Kobayashi et al. showed that in 39% of the patients with fistula formation, multiple neighboring organs were penetrated. However, 44% of those patients showed no stromal invasion histologically, and in 67%, the fistula was caused by mechanical penetration without cancer invasion [6]. Likewise, our patient’s fistula most likely occurred as a consequence of mechanical penetration from the excessive pressure of mucin-filled ducts and inflammation or autodigestion by the enzyme-rich fluids [14].

Decreasing mucinous pressure within the branch system and providing a mucinous outlet may potentially alleviate symptoms. For example. one case report commented on the resolution of the patient’s abdominal pain after finding a gastropancreatic fistula [15]. This could have explained our patient’s asymptomatic course if the development of a gastropancreatic fistula had occurred prior to her initial ED visit and contributed to the alleviation of her symptoms. Managing the decrease of excess mucus and providing adequate drainage had been difficult in our case. Decompressing the biliary system with percutaneous transhepatic biliary drainage with n-acetylcysteine three times daily for 10 days has been used [16]. If there is difficulty controlling drainage with stents in an IPMN fistulizing to CBD, like in our case, a choledochoduodenostomy is performed [17].

## Conclusions

Our case highlights the potential complications of excess mucin production from an unresected IPMN and shows an increasing burden of care for an elderly patient with dementia. Published literature shows universal failure when trying to decrease mucinous pressure with stents, especially after fistula formation.
